# Bibliometric analysis of current trends and emerging patterns in the application of nanomaterials for non-small cell lung cancer

**DOI:** 10.1007/s12672-025-02602-3

**Published:** 2025-05-18

**Authors:** Shanshan Cai, Weichang Yang, Hongquan Xing, Jiale Yang, Hongdan Luo, Xiaoqun Ye

**Affiliations:** 1 Department of Respiratory and Critical Care Medicine, The Second Affiliated Hospital, Jiangxi Medical College, Nanchang University, 1 Minde Road, Nanchang, 330006 Jiangxi People’s Republic of China; 2 Hepatobiliary and Pancreatic Surgery Division, Department of General Surgery, The 2nd Affiliated Hospital, Jiangxi Medical College, Nanchang University, Nanchang, 330038 China

**Keywords:** Bibliometric analysis, Non-small cell lung cancer, Nanomaterial, Research trend, Visualization

## Abstract

**Background:**

Lung cancer is one of the most prevalent tumor diagnoses and a leading cause of cancer-related mortality worldwide. Among its two primary subtypes, non-small cell lung cancer (NSCLC) accounts for approximately 80–85% of all lung cancer cases. Over the past decade, a significant number of publications have explored the application of nanomaterials in NSCLC. This study aimed to comprehensively evaluate the current state and evolving trends in research focused on utilizing nanomaterials as potent diagnostic and therapeutic tools for NSCLC.

**Methods:**

To identify all pertinent publications, we used the Web of Science Core Collection (WoSCC) database. Based on stringent inclusion and exclusion criteria, relevant publications were carefully selected. For the bibliometric and visual analyses, we employed VOSviewer (version 1.6.20), CiteSpace (version 6.1.6), and R-bibliometrix (version 4.3.2).

**Results:**

Our analysis encompassed 1880 studies that fulfilled the inclusion criteria. We observed a steady increase in annual publications from 2014 to June 22, 2024. China, the USA, and India have emerged as leading nations in this field. Notably, the Chinese Academy of Sciences and Wang J stood out as the most influential institutions and authors, respectively. Most publications are featured in The International Journal of Nanomedicine. The keywords used in these publications were closely tied to non-small cell lung cancer and nanomaterials. In the past three years, “green synthesis” exhibited the highest burst strength, while “immune response” and “nanocrystal” represented emerging areas of intense research interest.

**Conclusion:**

Through our exhaustive analysis, we synthesized the current research trends and emerging landscapes of nanomaterials in NSCLC. We characterized the publication patterns, pinpointed the most influential nations, institutions, authors, journals, and hot topics related to nanomaterial applications in NSCLC, and proposed potential avenues for future development.

## Introduction

Lung cancer is a pervasive malignancy, ranking as the primary cause of cancer-related deaths globally and imposing a formidable health challenge worldwide [1, 2]. In 2025, nearly 500 people will die from cancer every day, most of them from lung cancer, and it is estimated that lung cancer will accounts for approximately 124,730 deaths in the United States, continuing to be the leading cause of cancer mortality [3]. It holds the dubious distinction of being the second most common cancer among women and the most prevalent cancer among men worldwide. Among the two primary categories of lung cancer, non-small cell lung cancer (NSCLC) accounts for an overwhelming 80–85% of all cases, posing a formidable threat [4]. NSCLC is usually asymptomatic in its early stages, leading to late diagnosis accompanied by metastasis, which significantly contributes to its high mortality [5]. Unfortunately, the clinical performance of conventional anticancer drugs and diagnostic modalities has been unsatisfactory, likely because of factors such as a narrow therapeutic index, inadequate tumor targeting, and severe off-target toxicities [6]. In light of these challenges, applying nanomaterials and nano-enabled technologies has emerged as a promising avenue to enhance the efficacy of NSCLC diagnosis and treatment. These advancements have the potential to revolutionize the management of this devastating disease. The history of metal nanoparticles extends back to the fourteenth and thirteenth centuries BC, when Egyptians and Mesopotamians pioneered the use of metals in glassmaking, heralding the advent of the age of metal nanoparticles [7]. These ancient materials may well be the earliest practical examples of synthetic nanomaterials. Nanomaterials, which are characterized by their unique properties, have garnered extensive research interest because of their potential for accurate cancer diagnosis and effective treatment. Typically defined as materials with dimensions ranging from 1 to 1000 nm in at least one axis and a diameter within the 1–100 nm range [8], nanoparticles typically feature a concentrated drug core encapsulated within a functionalized outer layer or shell. These engineered nanoparticles undergo further optimization based on their size, shape, and surface characteristics to enhance their therapeutic efficacy and mitigate adverse effects [9]. Nanomaterials with diverse compositions offer promising avenues for developing effective NSCLC treatments.

Nanomaterials have emerged as a promising modality for the treatment of NSCLC. By serving as drug carriers, these materials facilitate targeted and precise drug delivery, significantly mitigating the side effects and drug resistance. Notably, anticancer drugs commonly employed in clinical settings are hydrophobic, posing challenges to their dissemination within aqueous environments [10]. Consequently, there is a pressing need for combination therapies that exhibit high efficacy and low toxicity to broaden the clinical benefits to a larger patient pool and enhance NSCLC prognosis. One strategic approach involves encapsulating hydrophobic drugs within nanoparticle carriers, which not only protects but also efficiently delivers hydrophilic drugs. This encapsulation method dramatically elevates the concentration of hydrophobic drugs in the human system by over 5 × 10^4^ times [11]. Furthermore, nanomaterials can be used as detection imaging agents, enabling the early diagnosis and precise localization of tumors or diseased tissues. Compared to traditional diagnostic methods, their minute size, excellent biocompatibility, and robust organ-targeting capabilities address the limitations of conventional clinical detection techniques [12] as well as their unique physical properties to empower innovative therapies for NSCLC, including photothermal/photodynamic, acoustodynamic, magnetothermal treatments, and even combination therapies. These advancements underscore the potential of nanomaterials as multifaceted solutions to the battle against NSCLC.

The utilization of nanomaterials in NSCLC treatment has immense potential to enhance traditional therapeutic approaches, offering heightened efficacy and reducing systemic adverse effects. Presently, a diverse array of innovative therapeutic formulations are under active investigation, each tailored with distinctive properties ideal for diverse NSCLC treatment scenarios. Several of these formulations have already secured clinical approval, while others are advancing through preclinical or initial clinical trial phases. Despite the escalating adoption of nanomedicine as an adjuvant treatment for NSCLC, there persist several obstacles that impede its practical application and full potential. These challenges encompass; but are not limited to, optimizing loading efficiency, achieving mass-production scalability, refining biodistribution patterns, elucidating pharmacokinetics, and mitigating toxicity concerns. Addressing and refining these factors are crucial to unlocking the full therapeutic potential of nanomaterials in NSCLC treatment.

Bibliometric analysis is a multidisciplinary field that employs mathematical and statistical techniques to quantitatively assess diverse media knowledge. This integrated knowledge system combines principles from mathematics, statistics, and philology, with a pronounced focus on quantification. Utilizing information visualization methods provides an insightful representation of a discipline's research progression, current status, emerging foci, and anticipated directions [13]. Notably, to the best of our knowledge, no published bibliometric analysis exists on the application of nanomaterials in NSCLC. Our study is a pioneering endeavor, the first of its kind to delve into the research trends of nanomaterials in NSCLC diagnosis and treatment over the past decade through a bibliometric analysis. We aim to summarize the developmental journey and contemporary research landscape of nanomaterials in this medical field while also proposing plausible directions for future advancements.

## Methods

### Search strategy

The Web of Science Core Collection (WoSCC) database served as the primary source for identifying all pertinent publications owing to its vast repository of scientific literature and its role as a primary source of general statistics for bibliometric software. Consequently, it is widely regarded as bibliometric research's most frequently utilized database [13, 14]. For this study, we retrieved and downloaded all relevant articles from the WoSCC database until June 22, 2024. Our search strategy for articles pertaining about nanomaterials and NSCLC entailed the use of a combination of keywords: "(non-small cell lung cancer OR non-small cell lung carcinoma OR NSCLC) AND (nano).” The search phrase "nano" allowed us to capture a broad range of terms commencing with "nano," encompassing nanoparticles, nanocarriers, nanomaterials, nanotechnology, nanocomposites, and other related concepts.

### Data extraction

The retrieved publications were systematically categorized into distinct file formats to facilitate analysis. From these publications, we extracted a comprehensive set of data including the title, author, affiliating institution, country of origin, journal title (along with the 2024 journal impact factor, IF), year of publication, citation count, and H-index. This meticulous data extraction process aimed to provide a nuanced understanding of the research landscape of nanomaterials and NSCLC.

### Data analysis

In our current research endeavor, we leveraged a trio of specialized tools—VOSviewer (version 1.6.20), CiteSpace (version 6.1.6), and R-bibliometric (version 4.3.2)—to conduct comprehensive bibliometric and visual analyses. VOSviewer was a software for constructing and viewing bibliometric maps and could display large bibliometric maps in an easy–to–interpret way and played a pivotal role in crafting visual maps that illuminated the most productive collaborations among countries, institutions, and authors, as well as in identifying the most cited journals and frequently co-occurring keywords. This tool enabled us to visualize the intricate network of research interactions, where each node represented a country, institution, author, or journal grouped according to their collaborative ties. The size of these nodes was proportional to the volume of publications, whereas the Total Link Strength (TLS) offered a quantitative measure of the overall level of cooperation within the network [14]. Concurrently, CiteSpace software was used to detect keywords and references with the strongest citation bursts, to construct visualization maps of co–cited references and keywords, and to plot a dual–map overlay of journals, which facilitated the construction of a timeline graph, revealing the historical evolution of the research field through co-cited references, and pinpointing the emergence of significant bursts of terms. This approach allowed us to trace the development of the key concepts and themes over time. Furthermore, in our keyword analysis, we employed rigorous criteria to exclude irrelevant keywords and merged them with similar meanings, thereby enhancing the clarity and focus of our findings. This step was crucial for gaining a more nuanced and accurate understanding of the research landscape surrounding nanomaterials and NSCLC. Finally, we utilized the H-index as a robust metric to evaluate the productivity and impact of authors, countries, and institutions, offering a quantitative assessment of their contributions to the field [15]. Through this multifaceted analysis, we aim to provide a comprehensive and insightful view of the current state of research on nanomaterials and NSCLC.

## Result

### Study selection and characteristics

**Fig. 1:**
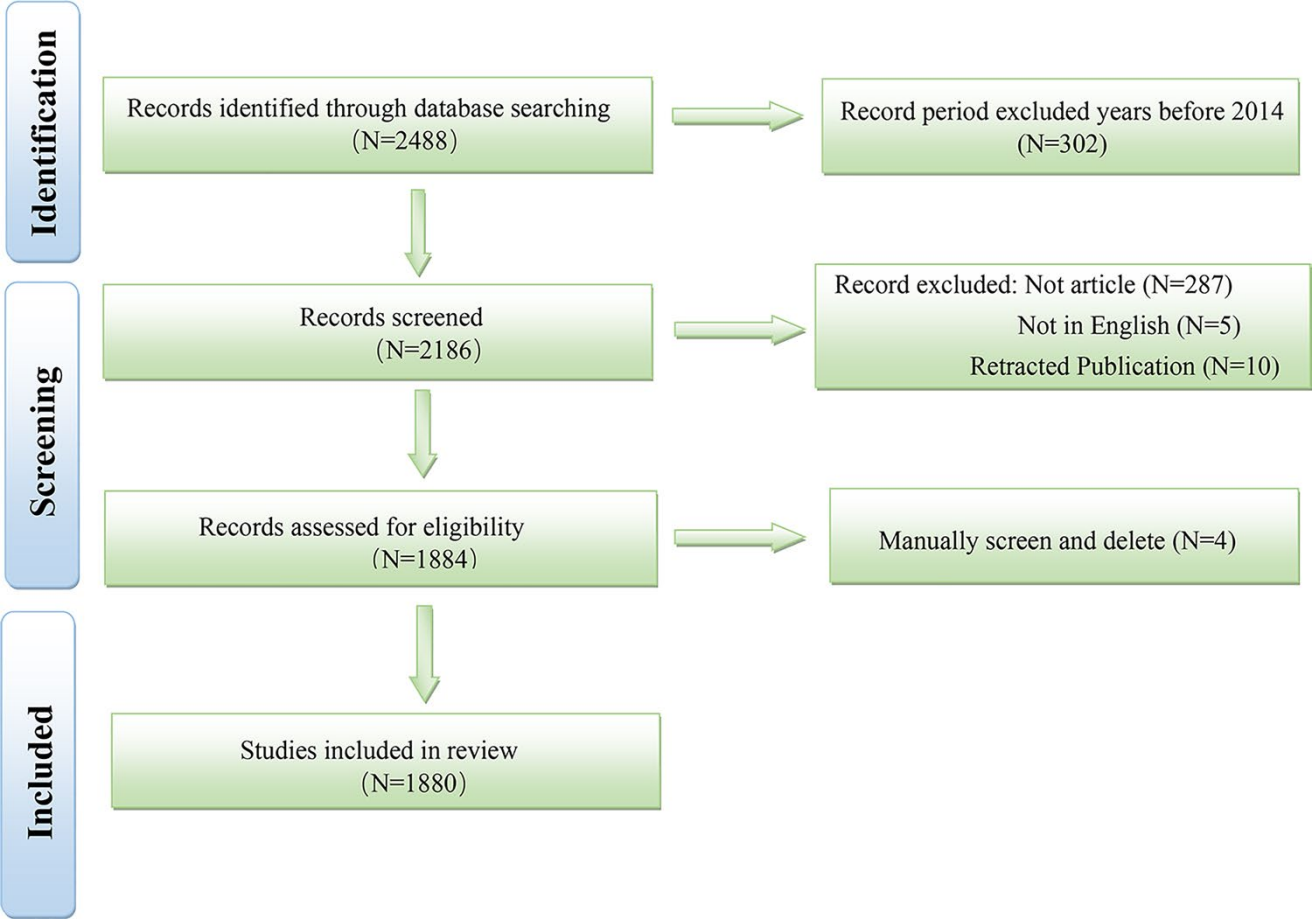
Flowchart of the literature screening process

As depicted in Fig. 1, a comprehensive search of the WoSCC database yielded 2488 publications, focusing on keywords about NSCLC and nanomaterials. Notably, the search was restricted to publications from 2014 onwards, excluding earlier years. During the initial screening phase, 5 publications were disqualified due to language barriers, 287 were excluded based on their publication types, and an additional 10 were omitted as they had been retracted. Subsequently, the titles and abstracts of the remaining 1884 publications were rigorously evaluated. Ultimately, 1880 studies that fulfilled the inclusion criteria were included in the analysis.

### Overview of the included publications

**Fig. 2:**
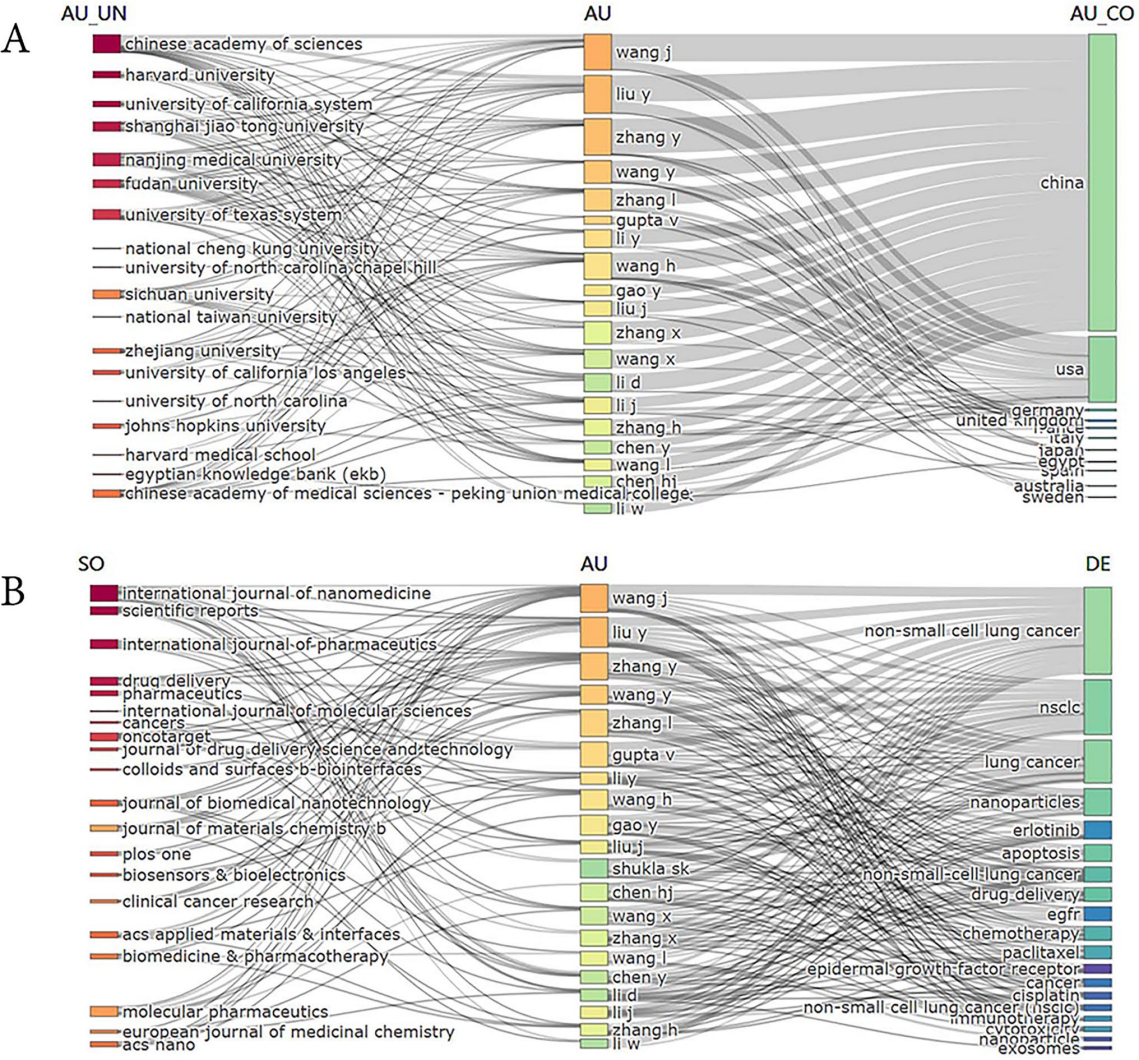
The three-Field Plot. **A** The 20 authors with the highest number of publications and related institutions, countries,** B** Journal and keywords

We created a three-field plot to visually represent the top 20 authors with the highest publication counts, along with their affiliated institutions, countries, journals, and keywords (Fig. 2). Notably, most of these prominent authors hailed from China and the USA. Specifically, the Chinese Academy of Sciences and Harvard University were the leading institutions in their respective countries, contributing the highest number of publications (Fig. 2A). The publications predominantly appeared in journals focused on nanomaterials, and the keywords used in these publications strongly correlated with lung cancer and nanomaterials (Fig. 2B).

**Fig. 3:**
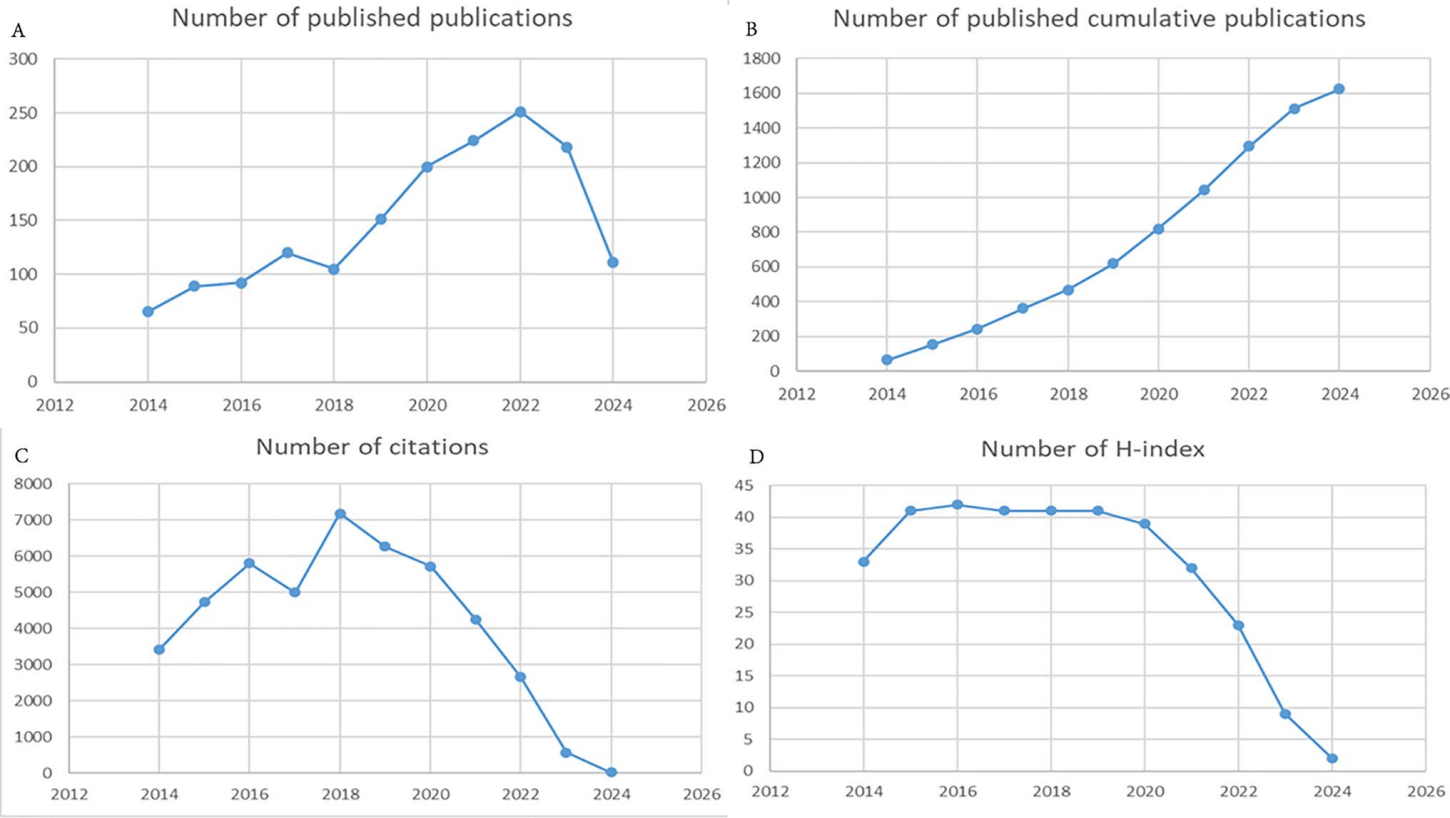
The characteristics of the included studies, **A** the number of annual publications; **B** the number of published cumulative publications; **C** the number of citations of the publications; **D** the number of H-index values of the publications

We have summarized the salient characteristics of the included studies. As depicted in Fig. 3A, the annual distribution of publications from 2014 to 2024 revealed a consistent upward trend, signifying a growing interest in the intersection of NSCLC and nanomaterials. The year 2022 witnessed a peak in publications with 251 contributions, accounting for 13.35% of the total. Similarly, the cumulative number of publications increased steadily from 2014 to 2024 (Fig. 3B). In terms of citations, there has been a considerable level of engagement, with more than 2000 citations annually from 2014 to 2022 (Fig. 3C). The H-index of the publications remained stable above 30 from 2014 to 2021, peaking in 2016, with an H-index of 42 (Fig. 3D).

### Analysis of country/region and institution attributes of the publications

**Fig. 4:**
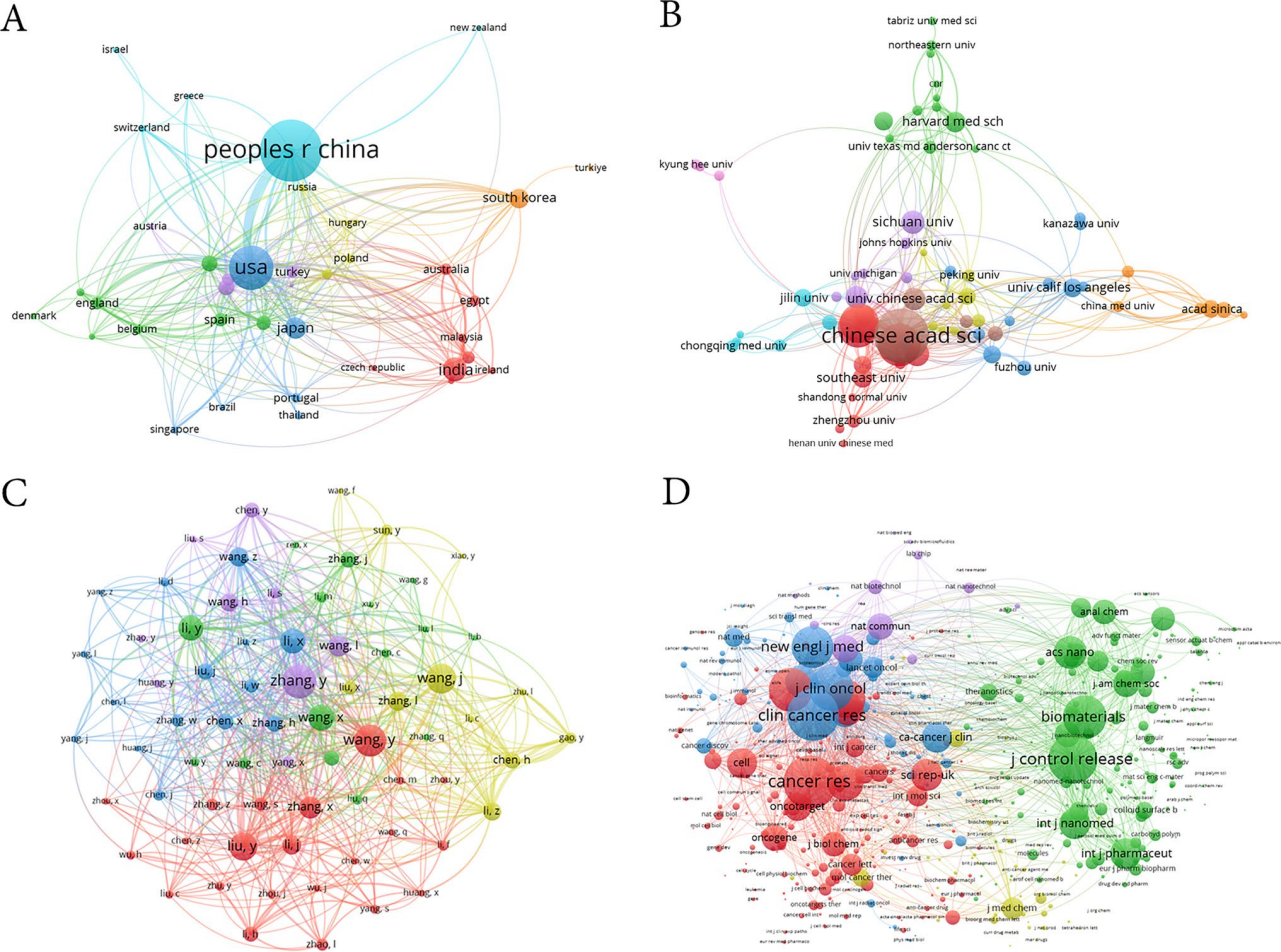
The coauthorship network map of **A** Countries** B** Institutions **C** Authors and **D** Journals

**Fig. 5:**
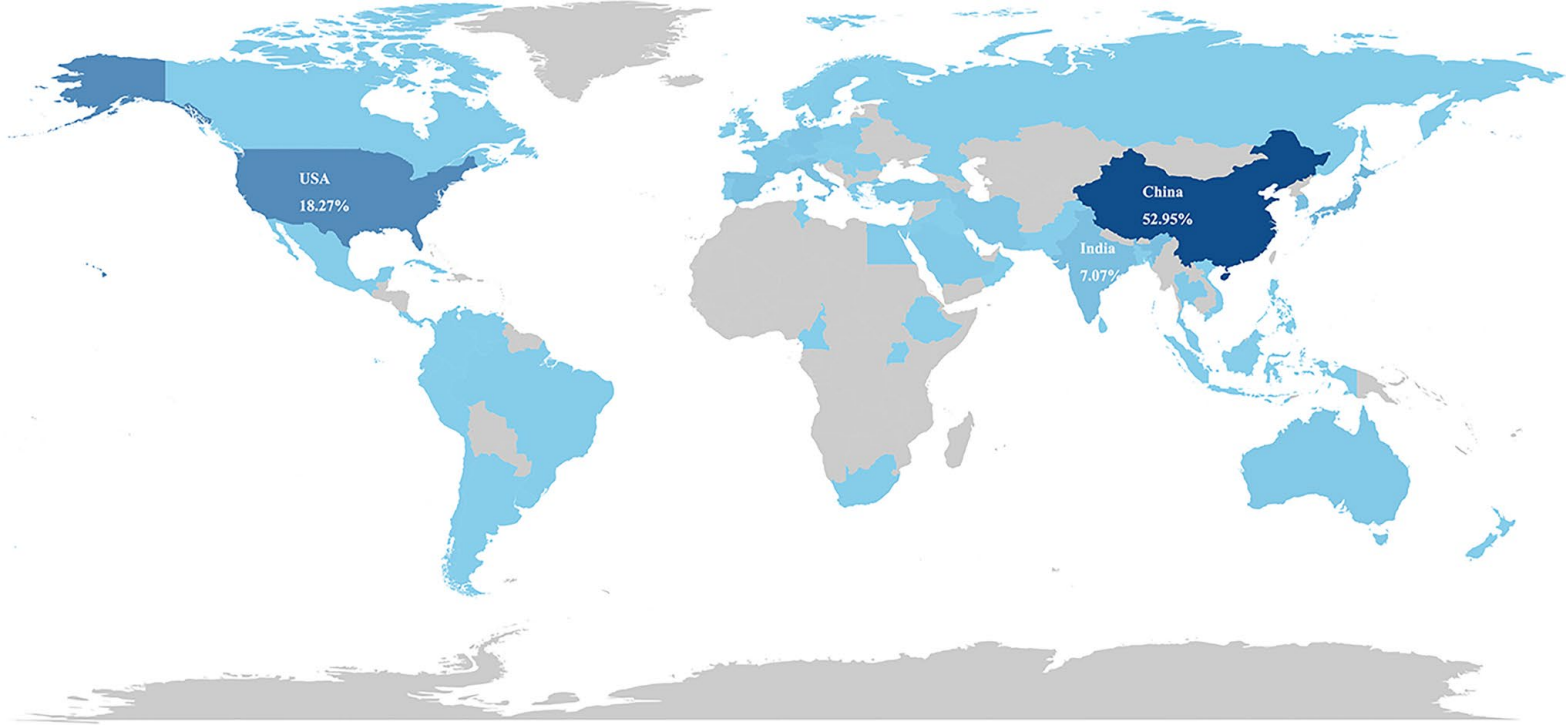
Country attributes of the publications

The visualization of the international co-authorship network among countries is depicted in Fig. 4A, including a total of 40 participating nations. Notably, China emerges as the pinnacle of international collaboration, boasting the strongest network with a TLS score of 186 and maintaining its closest ties with the USA. Focusing on quantitative achievements, we delved into the metrics of the top ten most prolific countries. As shown in Fig. 5 and Table 1, China led the pack with 861 publications, accounting for 45.80% of the total, closely followed by the USA with 297 publications (15.80%), and India, rounding out the top three with 115 publications (6.12%). Additionally, China reigns supreme in both citations, amassing 19,511 and the H-index, with a score of 162.

**Table 1:** The top 10 most productive countries regarding nanomaterial and NSCLC research from 2014 to 2025

Rank	Country	Counts	Percentage%	Total citation	H-Index
1	China	861	45.80	19,511	162
2	USA	297	15.80	9926	117
3	India	115	6.12	2205	44
4	Korea	77	4.10	1399	39
5	Japan	76	4.04	1031	41
6	Italy	40	2.13	891	44
7	Spain	33	1.76	3222	62
8	Iran	33	1.76	834	36
9	Germany	32	1.70	682	40
10	Turkey	31	1.65	521	24

Figure 4B presents the intricate co-authorship network among institutions, encompassing 72 distinguished entities. The Chinese Academy of Sciences stands out as the most extensive collaborative network, attaining a TLS score of 75. Zooming in on the top ten institutions by productivity, as summarized in Table 2, the Chinese Academy of Sciences claims the top spot with 145 publications, representing 7.71% of the total. Harvard University and the University of California System followed closely, with 114 and 101 publications, respectively, accounting for 6.06% and 5.37% of the total, respectively. Moreover, the Chinese Academy of Sciences not only leads in terms of output, but also excels in citations, garnering 3,036 total citations, and the H-index, where it achieves a score of 32.

**Table 2:** The top 10 most productive institutions regarding nanomaterial and NSCLC research from 2014 to 2025

Rank	Institutions	Country	Counts	Percentage%	Total citation	H-Index
1	Chinese Academy of Sciences	China	145	7.71	3036	32
2	Harvard University	USA	114	6.06	2521	21
3	University of California System	USA	101	5.37	1674	21
4	Shanghai Jiao Tong University	China	99	5.27	1190	22
5	Nanjing Medical University	China	97	5.16	1705	23
6	Fudan University	China	93	4.95	1530	23
7	University of Texas System	USA	92	4.89	1131	18
8	National Cheng Kung University	China	75	3.99	259	8
9	University of North Carolina	USA	72	3.83	858	16
10	University of North Carolina Chapel Hill	USA	72	3.83	858	16

### Analysis of authors of publications

A comprehensive analysis of the publications revealed a remarkable contribution from 12,416 authors. The top 10 authors with the most significant publication outputs are highlighted in Table 3. Wang J led the pack with 42 publications, accounting for 2.23% of the total, closely followed by Liu Y with 41 publications, and Zhang Y with 40. Liu Y not only excels in terms of output, but also stands out as the author with the highest total citations, totaling 1727, and the highest H-index of 19, reflecting their profound influence and impact within the field. To visualize the intricate web of collaborations among these authors, Fig. 4C presents an author cooperation network map. Within this intricate network, Gao Y emerged as the most interconnected node, boasting the highest number of collaborative relationships with other authors and achieving a TLS score 46.

**Table 3:** The top 10 productive authors regarding nanomaterial and NSCLC research from 2014 to 2025

Rank	Authors	Counts	Percentage%	Total citation	H-Index
1	Wang J	42	2.23	1076	19
2	Liu Y	41	2.18	1727	19
3	Zhang Y	40	2.13	792	18
4	Wang Y	26	1.38	765	15
5	Zhang L	26	1.38	600	16
6	Gupta V	20	1.06	530	13
7	Li Y	20	1.06	441	10
8	Wang H	20	1.06	427	13
9	Gao Y	19	1.01	486	12
10	Liu J	19	1.01	750	12

### Analysis of source journals and co-cited journals

Among the 273 articles published in the top ten journals (Table 4), constituting 14.52% of the total publications, the International Journal of Nanomedicine, Scientific Reports, and International Journal of Pharmaceutics stood out as the most prominent venues for research in this field. Notably, the International Journal of Nanomedicine led to the highest cumulative citation count (1368), whereas Oncotarget boasted the highest average citations per article (37.29).

**Table 4:** The top 10 most productive journals regarding nanomaterial and NSCLC research from 2014 to 2025

Rank	Journals	Counts	Percentage%	Total citation	Average citation	H-Index	IF (2024)
1	International Journal Of Nanomedicine	44	2.34	1368	31.09	24	6.6
2	Scientific Report	36	1.91	1001	27.81	17	3.8
3	International Journal Of Pharmaceutics	30	1.60	655	21.83	15	5.3
4	Drug Delivery	29	1.54	751	25.9	16	6.5
5	Pharmaceutics	28	1.49	378	13.5	10	4.9
6	International Journal Of Molecular Sciences	27	1.44	316	11.7	9	4.9
7	Cancers	26	1.38	321	12.35	11	4.5
8	Oncotarget	24	1.28	895	37.29	18	NA
9	Journal Of Drug Delivery Science And Technology	22	1.17	253	11.50	9	4.5
10	Colloids And Surfaces B-Biointerfaces	21	1.12	377	17.95	11	5.4

Regarding the H-index, a measure of a journal's research impact, the International Journal of Nanomedicine ranked first at 24, closely followed by Scientific Reports at 17. The Impact Factor (IF), a crucial metric assessing a journal's significance and the quality of its publications, placed the International Journal of Nanomedicine at the forefront with 6.6, narrowly surpassed by Drug Delivery with 6.5.

The journal cocitation network map (Fig. 4D) revealed Cancer Research, Clinical Cancer Research, and Journal of Controlled Release as the top three most cocited journals, with citation counts of 1516, 1442, and 1344, underscoring their influence and relevance in the field.

### Analysis of highly cited studies

**Fig. 6:**
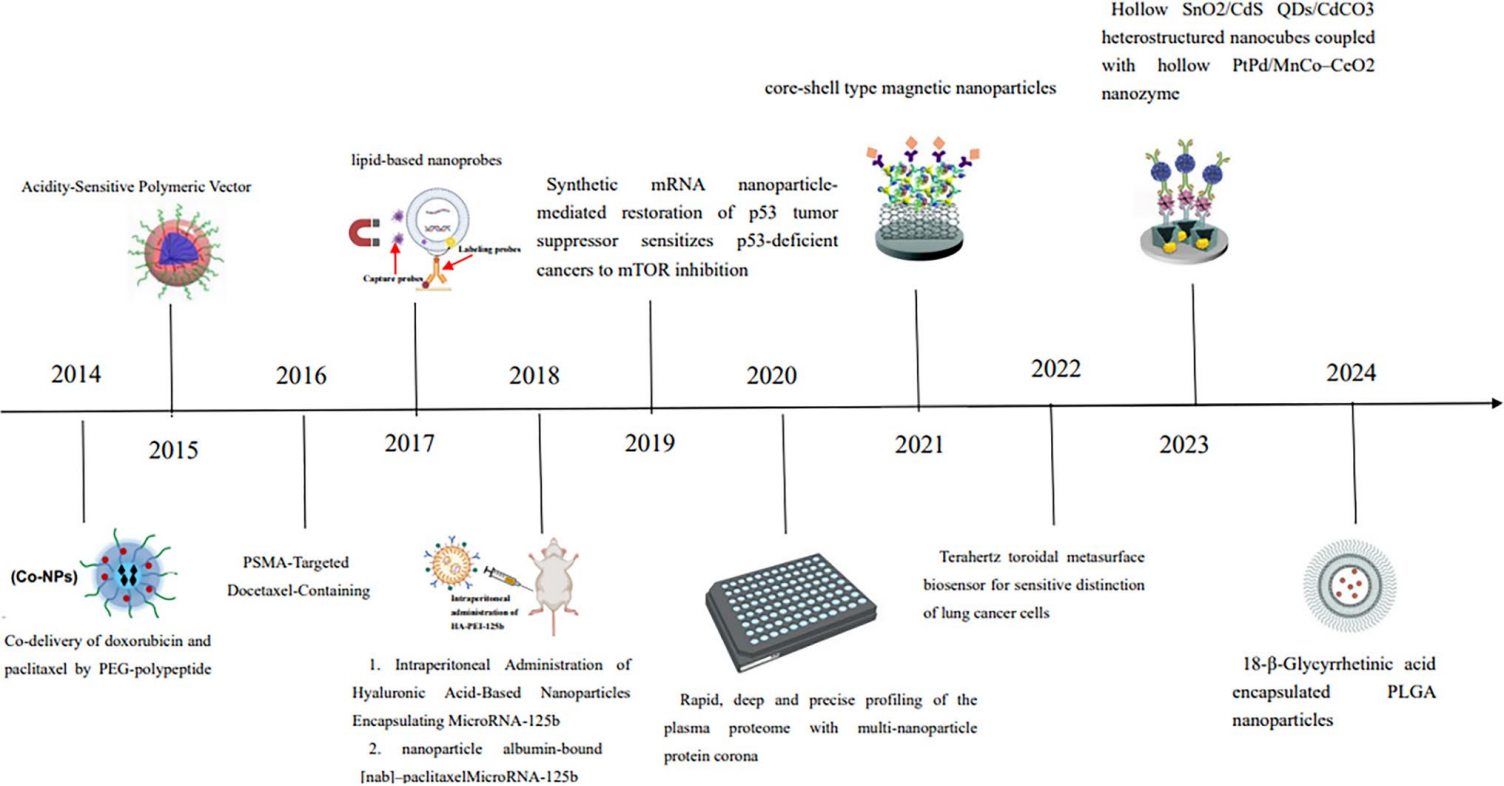
Timeline of the most cited publications on NSCLC and nanomaterials from 2014 to 2024

Figure 6 shows the nanomaterials that were the focus of the most frequently cited publications each year during the study period. This visualization provides insights into evolving trends and preferences in nanomaterial research over time [16–27]. These studies frequently used nanoparticles and nanocarriers. The ten most cited studies are detailed in Table 5. These highly cited studies into following common themes:

**Table 5:** The top 10 most cited references regarding nanomaterial and NSCLC research from 2014 to 2025

Rank	Title	Year	Institution	Author	Journal (IF 2024)	Citation
1	Pembrolizumab plus Chemotherapy for Squamous Non-Small-Cell Lung Cancer	2018	Spanish National Cancer Research Center, Spanish	Luis Paz-Ares, Alexander Luft, David Vicente, etal	New England Journal of Medicine (IF = 96.2)	2419
2	Co-delivery of doxorubicin and paclitaxel by PEG-polypeptide nano-vehicle for the treatment of non-small cell lung cancer	2014	Key Laboratory of Polymer Ecomaterials, Changchun Institute of Applied Chemistry, University of Chinese Academy of Sciences, China	LV S, Tang Z, Li M, etal	Biomaterials (IF = 12.8)	299
3	Tumor Acidity-Sensitive Polymeric Vector for Active Targeted siRNA Delivery	2015	The CAS Key Laboratory of Innate Immunity and Chronic Disease, China	CY Sun, S Shen, CF Xu,etal	Journal of the american chemical society (IF = 14.4)	296
4	Rapid magnetic isolation of extracellular vesicles via lipid-based nanoprobes	2017	Micro & Nano Integrated Biosystem (MINIBio) Laboratory, The Pennsylvania State University, University Park, U.S.A	Wan Y, Cheng G, Liu X, etal	Nature Biomedical Engineering (IF = 26.8)	192
5	Phase I Study of PSMA-Targeted Docetaxel-Containing Nanoparticle BIND-014 in Patients with Advanced Solid Tumors	2016	Translational Genomic Research Institute and Virginia G, USA	Von Hoff DD, Mita MM, Ramanathan RK, etal	Clinical Trial (IF = 10.0)	187
6	Repolarization of Tumor-Associated Macrophages in a Genetically Engineered Non-small Cell Lung Cancer Model by Intraperitoneal Administration of Hyaluronic Acid-Based Nanoparticles Encapsulating MicroRNA-125b	2018	Department of Pharmaceutical Sciences, School of Pharmacy, USA	Parayath NN, Parikh A, Amiji MM, etal	Nano Letters (IF = 9.6)	186
7	Synthetic mRNA nanoparticle-mediated restoration of p53 tumor suppressor sensitizes p53-deficient cancers to mTOR inhibition	2019	Brigham and Women's Hospital, Harvard Medical School, USA	Kong N, Tao W, Ling X, etal	Science Translational Medicine (IF = 15.8)	175
8	Rapid, deep and precise profiling of the plasma proteome with multi-nanoparticle protein corona	2020	Broad Institute of MIT and Harvard, Cambridge, MA, USA	Blume JE, Manning WC, Troiano G, etal	Observational Study (IF = 14.7)	165
9	Nanofiber membrane supported lung-on-a-chip microdevice for anti-cancer drug testing	2018	Materials Genome Institute, Shanghai University, China	Yang X, Li K, Zhang X, etal	Lab on a Chip (IF = 6.1)	164
10	Temperature-Responsive Multilayer Films of Micelle-Based Composites for Controlled Release of a Third-Generation EGFR Inhibitor	2020	Jiangsu University, Institution Life Sciences, Jiangsu, China	Xu, L; Wang, HL, Chu, ZH, etal	ACS Applied Polymer Materials (IF = 4.4)	163

#### Drug delivery

Several highly cited studies focused on the use of nanomaterials for drug delivery in NSCLC. These include a study by LV et al. titled "Co-delivery of doxorubicin and paclitaxel by PEG-polypeptide nano-vehicle for the treatment of non-small cell lung cancer," which was published in BIOMATERIALS in 2014. This study demonstrated the effectiveness of a nano-vehicle for co-delivering two chemotherapeutic agents, doxorubicin and paclitaxel, for the treatment of NSCLC [18]. Another notable study in this area is "Temperature-Responsive Multilayer Films of Micelle-Based Composites for Controlled Release of a Third-Generation EGFR Inhibitor," by Xu et al., published in ACS Applied Polymer Materials in 2020. This study explored the use of temperature-responsive multilayer films for controlled drug release, specifically targeting EGFR in NSCLC [28].

#### Gene therapy

Research on gene therapy using nanomaterials for NSCLC was also well-represented in the highly cited studies. One such study is "Tumor Acidity-Sensitive Polymeric Vector for Active Targeted siRNA Delivery" by Sun et al., published in Journal of the American Chemical Society in 2015. This study developed a polymeric vector that is sensitive to tumor acidity for the active targeted delivery of siRNA to NSCLC cells [22]. Another study in this theme is "Repolarization of Tumor-Associated Macrophages in a Genetically Engineered Non-small Cell Lung Cancer Model by Intraperitoneal Administration of Hyaluronic Acid-Based Nanoparticles Encapsulating MicroRNA-125b" by Parayath et al., published in Nano Letters in 2018. This study investigated the use of hyaluronic acid-based nanoparticles encapsulating microRNA-125b for the repolarization of tumor-associated macrophages in a genetically engineered NSCLC model [19].

#### Diagnostics

While the majority of the highly cited studies focused on drug delivery and gene therapy, a few studies explored the use of nanomaterials for diagnostics in NSCLC. One such study is "Rapid, deep and precise profiling of the plasma proteome with multi-nanoparticle protein corona" by Blume et al., published as an observational study in 2020. This study demonstrated the use of multi-nanoparticle protein corona for rapid, deep, and precise profiling of the plasma proteome, which could have potential applications in the early detection of NSCLC [16]. Another study in this area is "Rapid magnetic isolation of extracellular vesicles via lipid-based nanoprobes" by Wan et al., published in Nature Biomedical Engineering in 2017. This study developed a method for the rapid magnetic isolation of extracellular vesicles using lipid-based nanoprobes, which could be useful for diagnostic purposes in NSCLC [24].

### Analysis of co-cited references

**Fig. 7:**
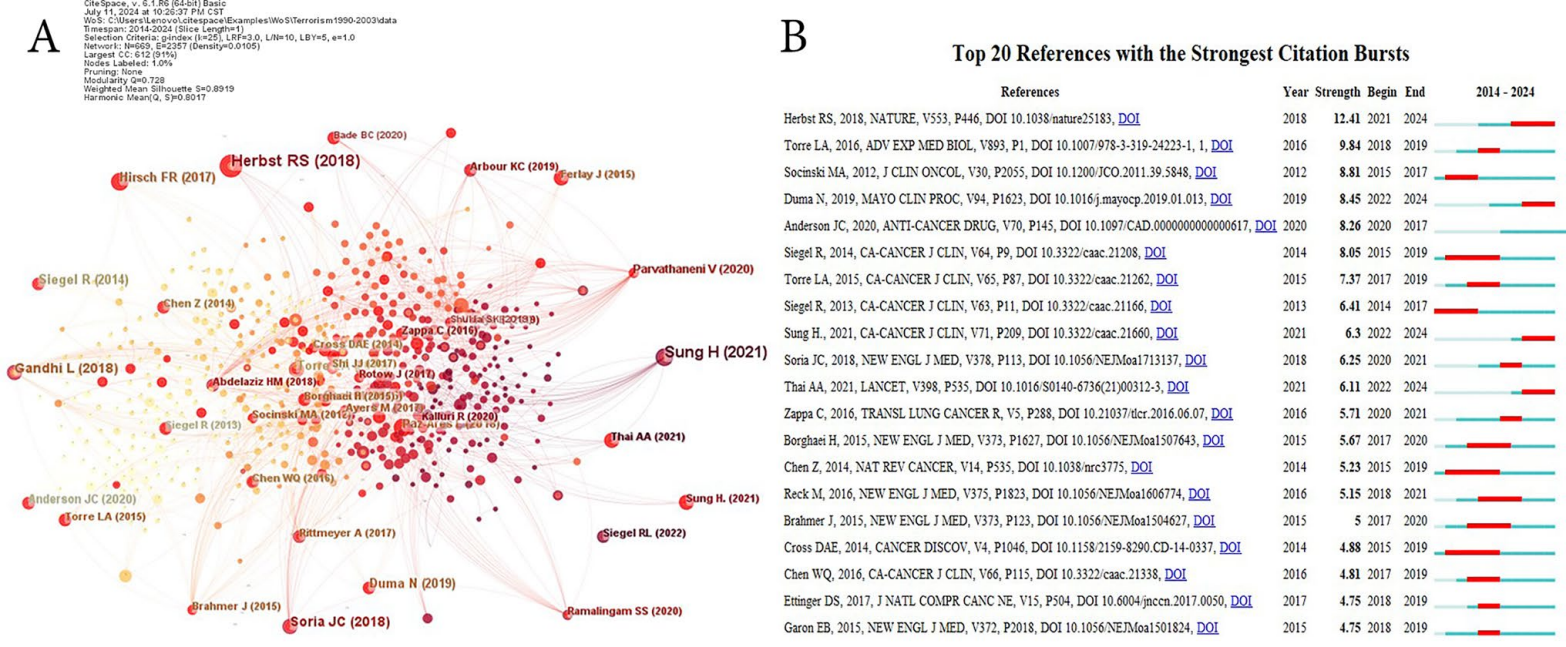
**A** The visualization of the co-cited publications; **B** the top 20 references with the strongest citation bursts

Table 6 highlights the top 10 most cited publications related to nanomaterials and NSCLC, with a visual representation of co-cited publications achieved using CiteSpace software. Notably, the publication by Professor Roy S. Herbst and Daniel Morgensztern from the USA, centering on novel drugs and combination therapies for NSCLC, garnered the highest number of citations, totaling 90 [29], Among the top 10 co-cited publications (Fig. 7A), "A Cancer Journal for Clinicians" stands out with the highest impact factor (IF 2024 = 503.1), closely followed by "New England Journal of Medicine" (IF 2024 = 96.2). To further emphasize the dynamic nature of citation patterns, we leveraged CiteSpace to identify the top 20 references experiencing significant citation bursts. A citation burst signifies a surge in attention towards a particular reference over a defined time period. As depicted in Fig. 7B, the most prominent citation burst stems from Herbst RS's 2018 paper [29], closely followed by Torre LA et al.'s 2016 contribution in "Advances In Experimental Medicine And Biology" [30], and Socinski MA et al.'s 2012 article in "JOURNAL OF CLINICAL ONCOLOGY" [31]. These findings underscore the significant impact and relevance of these publications in the field.

**Table 6:** Top 10 co-cited publications

Rank	Title	Year	Institution	Author	Journal (IF 2024)	Co-citation
1	The biology and management of non-small cell lung cancer	2018	Yale Cancer Center, Yale School of Medicine, New Haven, Connecticut, USA	Roy S. Herbst, Daniel Morgensztern & Chris Boshoff, etal	Nature (IF = 50.5)	90
2	Global Cancer Statistics 2020: GLOBOCAN Estimates of Incidence and Mortality Worldwide for 36 Cancers in 185 Countries	2021	Surveillance and Health Equity Science, American Cancer Society, Atlanta, Georgia, USA	Hyuna Sung, Jacques Ferlay MSc, Rebecca L. Siegel, etal	A Cancer Journal for Clinicians (IF = 503.1)	59
3	Pembrolizumab plus Chemotherapy in Metastatic Non-Small-Cell Lung Cancer	2018	From NYU Perlmutter Cancer Center, New York, USA	Leena Gandhi, Delvys Rodríguez-Abreu, Shirish Gadgee, etal	New England Journal of Medicine (IF = 96.2)	32
4	Osimertinib in Untreated EGFR-Mutated Advanced Non-Small-Cell Lung Cancer	2018	Gustave Roussy Cancer Campus and University Paris-Sud, Orsay, France	Jean-Charles Soria, Yuichiro Ohe, Johan Vansteenkiste, etal	New England Journal of Medicine (IF = 96.2)	30
5	Lung Cancer Statistics	2016	Surveillance and Health Services Research, Intramural Research Department, American Cancer Society, USA	Lindsey A Torre, Rebecca L Siegel, Ahmedin Jemal, etal	Advances In Experimental Medicine And Biology (NA)	29
6	Non–Small Cell Lung Cancer: Epidemiology, Screening, Diagnosis, and Treatment	2019	Division of Medical Oncology, Department of Medicine, University of Washington, Seattle	Narjust Duma, Rafael Santana-Davila, Julian R Molina, etal	Mayo Clin Proc (IF = 6.9)	25
7	Cancer Statistics, 2014	2014	Surveillance and Health Equity Science, American Cancer Society, Atlanta, Georgia, USA	Rebecca L Siegel, Kimberly D Miller, Nikita Sandeep Wagle, etal	A Cancer Journal for Clinicians (IF = 503.1)	23
8	Lung cancer: current therapies and new targeted treatments	2017	Department of Medicine, Division of Medical Oncology and Department of Pathology, University of Colorado Cancer Center, Denver, USA	Fred R Hirsch, Giorgio V Scagliotti, James L Mulshine, etal	Lancet (IF = 98.4)	23
9	Pembrolizumab plus Chemotherapy for Squamous Non–Small-Cell Lung Cancer	2018	Hospital Universitario Virgen Macarena, Seville, Spain	Luis Paz-Ares, Alexander Luft, David Vicente, M, etal	New England Journal of Medicine (IF = 96.2)	22
10	Pembrolizumab versus Chemotherapy for PD-L1–Positive Non–Small-Cell Lung Cancer	2016	Lung Clinic Grosshansdorf, Airway Research Center North, German Center of Lung Research, Grosshansdorf, Germany	Martin Reck, Delvys Rodríguez-Abreu, Andrew G Robinson, etal	New England Journal of Medicine (IF = 96.2)	19

### Keyword analysis of research hotspots

**Fig. 8:**
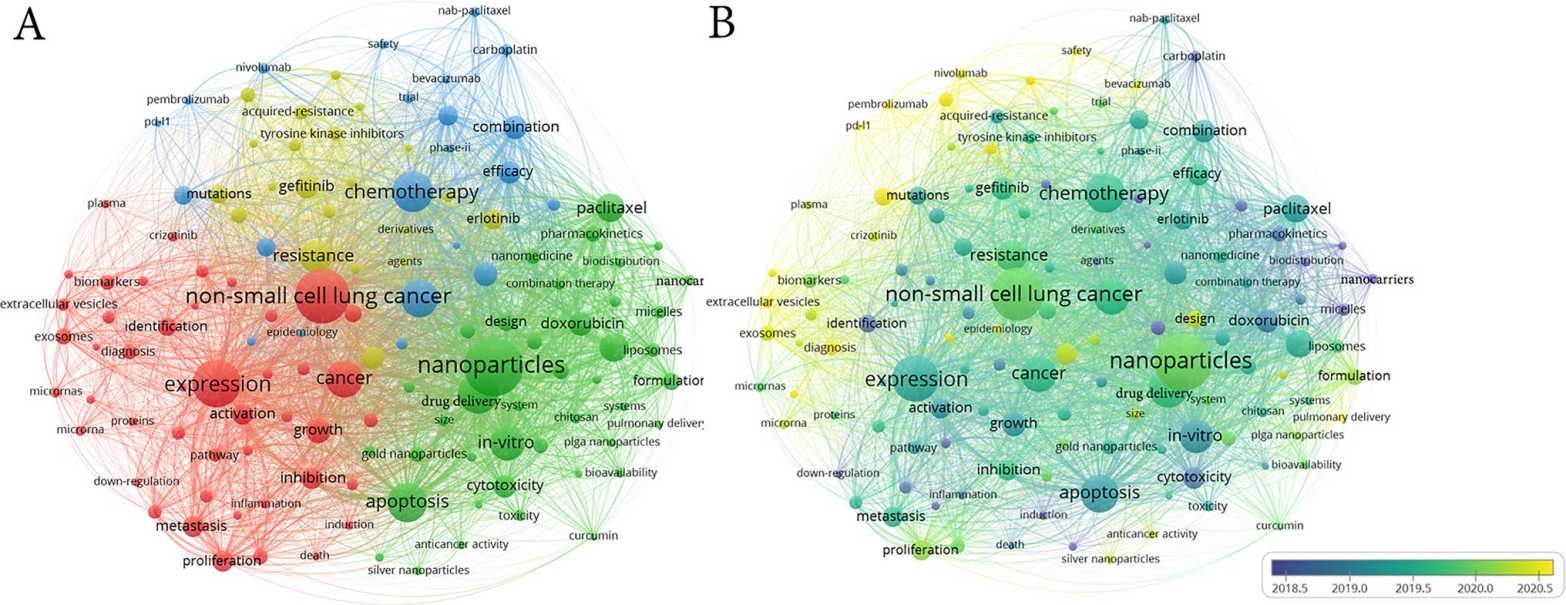
**A** The network and** B** overlay visualization maps of co-occurring keywords

Keyword co-occurrence analysis offers a compelling means of identifying the prevalent research themes within a field. As shown in Fig. 8, the intricate web and overlapping visualizations of simultaneously appearing keywords offer a compelling visual representation of the interconnected research domains. The top 10 most prevalent keywords reveal a focus on "non-small-cell lung cancer," "nanoparticles," and their applications in drug delivery, apoptosis, chemotherapy, and the mechanisms underpinning drug resistance and NSCLC mutations. These keywords were clustered into four distinct groups, each emphasizing specific aspects of the research landscape (Fig. 8A). The prominent red cluster emphasizes the mechanisms underlying NSCLC metastasis and growth, whereas the green cluster highlights nanocarriers and loaded agents for pulmonary delivery and combination therapies. The blue cluster focuses on clinical trials of nanoparticles in chemotherapy, whereas the yellow cluster focuses on drug resistance in NSCLC.

Temporal trends in keyword usage are evident in Fig. 8B, with earlier publications (purple) and recent developments (bright yellow). For instance, keywords such as "nanocarriers", “gold nanoparticles”, “stem cells” and “polymer micelles” were early topics. Conversely, terms such as “PLGA nanoparticles” and “pulmonary delivery” emerged later during the study period.

**Fig. 9:**
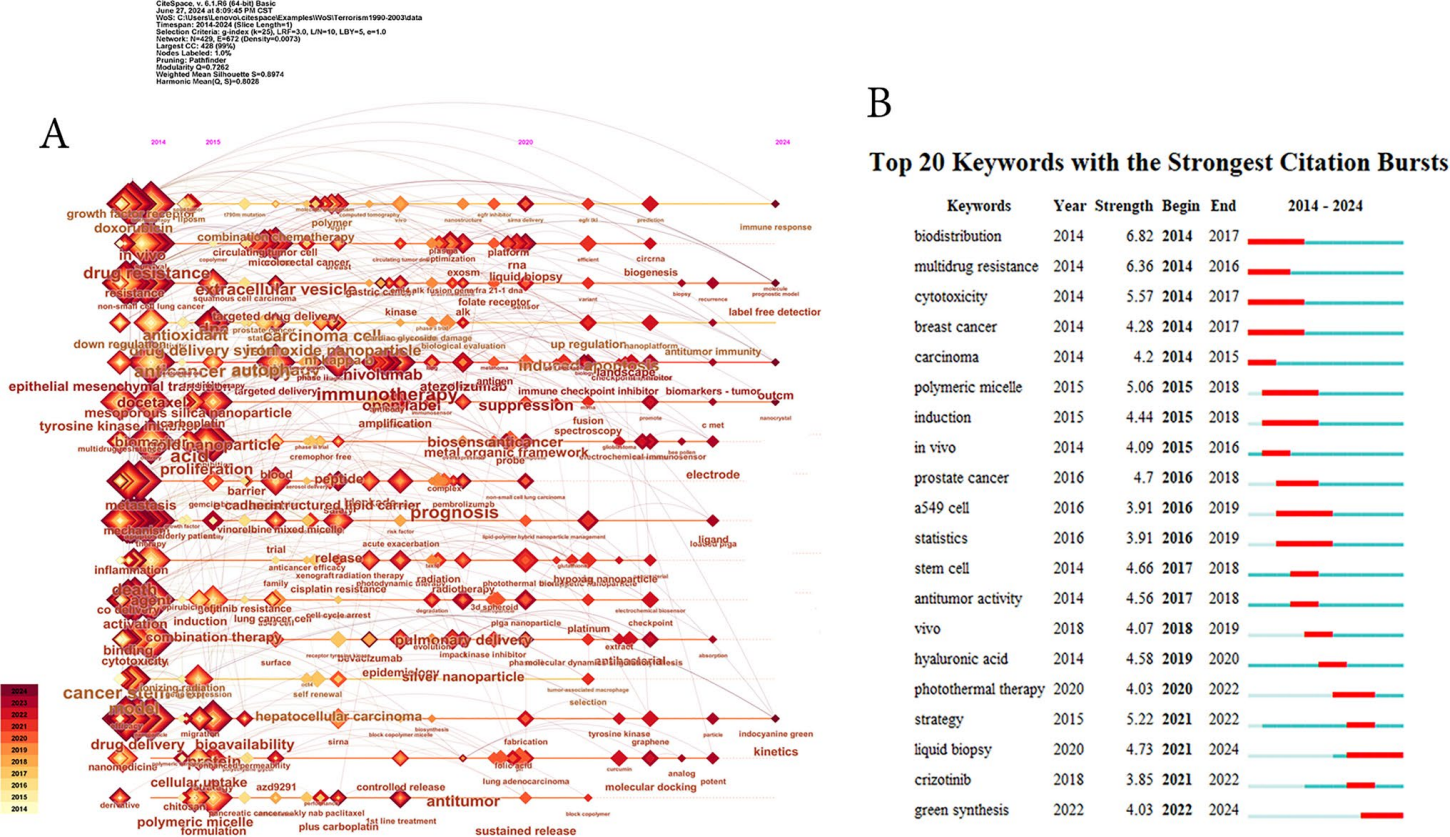
**A** The keyword evolution over time; **B** the top 20 keywords with the strongest citation bursts

The keyword evolution shown in Fig. 9A reveals shifts in research interest over time. Pre-2015, hot topics centered on NSCLC, drug delivery, and co-delivery. From 2015 to 2020, there has been a surge in interest in pulmonary delivery, gold nanoparticles, and silver nanoparticles (AgNPs). Post-2020, new hotspots have emerged, including lipid-polymer hybrid nanoparticles, molecular docking, and nanoplatforms. Notably, the "immune response" and "nanocrystal" began to gain traction in 2024.

Moreover, the strength of keyword bursts in Fig. 9B serves as a gauge of the dynamism and novelty of the research fronts. Among the top 20 bursty keywords, "green synthesis" stood out, with the highest burst strength in recent years, indicative of its growing popularity. In addition, the continued bursts of "green synthesis" and "liquid biopsy" in 2024 signify their enduring relevance as research hotspots.

## Discussion

To the best of our knowledge, this study marks the inaugural comprehensive bibliometric analysis of publications concerning the application of nanomaterials in non-small cell lung cancer (NSCLC), spanning the period from 2014 to 2024. Our findings revealed a pronounced upward trend in the annual number of publications within this domain, culminating in the peak year of 2022. Notably, 2018 witnessed the highest volume of citations, likely attributed to the seminal work by Luis Paz-Ares et al. published that year [21], which significantly impacted the field. Although the H-index of publications remained largely stable above 30 from 2014 to 2021, the decline in both citations and the H-index over the past four years could be attributed to the proximity of the data collection period, newly published articles often take months or even years to accumulate enough citations, so they have less impact on the H-index in the early stages of data collection. Compared with older, widely cited articles, newer articles have a weaker impact on the H-index due to fewer citations, resulting in a temporary decline in the index. The decline in the H-index does not indicate a change in research quality or academic impact. Instead, as these new articles accumulate over time, their citations will gradually grow, and the H-index is expected to pick up over time. Therefore, the current H-index decline is only due to time constraints, rather than a true reflection of research influence.

Subsequently, we analyzed the most influential nations, institutions, authors, and journals within this research area. Both China and the USA have made substantial contributions, yet China has emerged as the dominant force underscored by its leadership in international collaborations, publications, citations, and the H-index. Furthermore, the most productive institution and author hail from China, the Chinese Academy of Sciences and Wang J, respectively, occupied the top spots. The top 10 journals, predominantly focused on nanomaterials, exhibit an average impact factor exceeding 5, with the International Journal of Nanomedicine (IF = 6.6) standing out owing to its unparalleled number of publications, citations, and H-Index, solidifying its significance in this field.

Publications that garner the highest number of citations are universally regarded as the most pivotal and influential contributions within the field. Consequently, we conducted an analysis of the top ten most-cited publications. Notably, six of these publications focused on the utilization of nanocarriers for the delivery of drugs or genes for cancer treatment, reflecting the prevalent and compelling nature of this research topic within the discipline. Among these, the most frequently cited work by Paz-Ares et al., which also pertained to nanocarriers, corroborated that the integration of pembrolizumab into standard chemotherapy regimens comprising carboplatin and nab-paclitaxel significantly prolonged both overall survival and progression-free survival among patients with previously untreated metastatic non-small cell lung cancer (NSCLC) [21]. The publication emphasized that the nanomaterial nab-paclitaxel combines paclitaxel with albumin through nanotechnology, resulting in nanoparticles with a precise particle size of approximately 130 nm. The unique properties of nab-paclitaxel confer superior therapeutic efficacy to chemotherapy drugs. Its ability to more efficiently induce immunogenic tumor death while preserving the vitality of normal immune cells underscores its advantageous position in antitumor therapy. The second most highly cited publication by Lv et al. [18] showed the remarkable antitumor efficacy of DOX + PTX co-delivered nanoparticles (Co-NPs), which significantly reduced tumor size compared to the free drug combination or single drug-loaded nanoparticles without eliciting any notable side effects during treatment. The third highly cited work by Sun et al. introduced a straightforward acid-sensitive bridged copolymer designed for the tumor-targeted systemic delivery of siRNA. The resulting nanoparticles exhibited prolonged circulation and efficient accumulation within tumor cells, leading to a safe and enhanced inhibition of NSCLC growth [22]. Clinical research conducted by Von Hoff DD focused on a novel tumor prostate-specific membrane antigen (PSMA)-targeted nanoparticle, BIND-014. The study concluded that BIND-014 was generally well tolerated and exhibited predictable and manageable toxicity profiles, along with a unique pharmacokinetic profile compared to conventional docetaxel in clinical practice [23]. Parayath et al. developed macrophage-specific HA-PEI nanoparticles that successfully transfected tumor-associated macrophages (TAMs) into the lung tissues of an NSCLC mouse model, highlighting their significant potential in anticancer immunotherapy [19]. Kong et al. suggested that the restoration of tumor suppressors through a synthetic mRNA nanoparticle delivery strategy could be synergistically combined with other therapies for a potent combinatorial approach to cancer treatment [17].

Furthermore, four additional studies emphasized the application of nanomaterials in the diagnosis and treatment of NSCLC. Wan et al. presented a lipid nanoprobe (LNP) for the rapid isolation of nanoscale extracellular vesicles, showing its efficiency and versatility that paves the way for point-of-care NSCLC diagnostics [24]. Blume et al. devised a highly parallel protein quantification platform that harnesses multi-nanoparticle protein coronas for in-depth proteomic sampling and biomarker discovery in NSCLC [16]. Yang et al. introduced a lung-on-a-chip utilizing a poly(lactic-co-glycolic acid) (PLGA) electrospun nanofiber membrane as the chip substrate and cellular scaffold. This innovative lung-on-a-chip platform is straightforward, efficient, and user-friendly, with anticipated significant applications in personalized NSCLC treatment and potential contributions to other clinical therapies and tissue engineering endeavors [32]. Xu et al. employed temperature-responsive micelles and hyaluronic acid biopolymers to construct a sandwich-like membrane via layer-by-layer (LBL) self-assembly technology based on hydrogen bonding, offering insights into the modification of conventional NSCLC drug delivery materials [28]. The top ten most highly cited publications underscore the pivotal role that nanomaterials can play in NSCLC diagnosis and treatment, contingent upon the appropriate optimization of their structure and function.

The keyword analysis of research hotspots showed that the most frequently occurring keywords were related to the nanoparticle-based treatment of NSCLC and mechanisms of tumor metastasis and growth, indicating that they were the most widely researched fields. In the network visualization diagram, all keywords were divided into the following four clusters: mechanism of NSCLC metastasis and growth, nanocarriers and loaded agents, clinical trials of nanoparticles, and drug resistance in NSCLC, which are the main topics explored in the research area. From the overlay map and the main hot research keywords, we found that nanomaterials and nanotechnology have made great progress in the diagnosis and treatment of NSCLC during the past 10 years.

In the early stage (Before 2015), researchers explored the use of nanomaterials for drug delivery in NSCLC. One notable example is the co-delivery of doxorubicin and paclitaxel by a PEG-polypeptide nano-vehicle, which was reported in 2014 [18]. This study demonstrated the feasibility of using nanomaterials to deliver multiple therapeutic agents simultaneously, enhancing the efficacy of treatment while potentially reducing side effects. However, early nanomaterials faced challenges related to stability and targeting efficiency. The PEG-polypeptide nano-vehicle was designed to address these issues by providing a stable platform for drug delivery and targeting specific cells through surface modifications. In Mid-Stage (2015 to 2020), as the field progressed, researchers began to focus on improving the design of nanoparticles to enhance their stability and targeting efficiency. One significant advancement was the development of tumor acidity-sensitive polymeric vectors for active targeted siRNA delivery, reported in 2015 [22]. These vectors were designed to release their payload in the acidic tumor microenvironment, improving targeting efficiency and reducing off-target effects. Additionally, rapid magnetic isolation of extracellular vesicles via lipid-based nanoprobes, reported in 2017 [24], demonstrated the potential of using nanomaterials for the isolation and analysis of biomarkers related to NSCLC progression. During this stage, researchers also began to explore the use of nanocrystals for drug delivery. Nanocrystals offer several advantages over traditional formulations, including improved solubility, stability, and bioavailability. However, their application in NSCLC was still in the early stages, and further research was needed to optimize their design and targeting efficiency. In recent years (after 2020), the field has seen a surge in research focused on the application of nanocrystals and molecular docking techniques in NSCLC treatment. Nanocrystals have emerged as a promising platform for delivering therapeutic agents with high stability and targeting efficiency. For example, a study reported in 2024 demonstrated the promising potential of the prepared cetuximab-functionalized phospholipid-coated paclitaxel nanocrystals in lung cancer [33]. This approach provided a new therapeutic option for patients with NSCLC. In addition to nanocrystals, molecular docking techniques have also played a crucial role in the evolution of nanotechnology-based treatments for NSCLC [34]. These techniques allow researchers to predict the binding affinity and orientation of therapeutic agents to their targets, enabling the design of more effective nanomaterials. By combining nanocrystals with molecular docking, researchers can optimize the design of nanomaterials for specific tumor types, further enhancing their efficacy and reducing side effects. In conclusion, the evolution of research trends in the application of nanomaterials for NSCLC has been marked by significant advancements and overcoming of various challenges. From early drug delivery trials to the current focus on nanocrystals and molecular docking techniques, researchers have continuously worked to improve the stability, targeting efficiency, and therapeutic efficacy of nanomaterials. By examining the specific innovations and how they addressed earlier constraints, we can better understand the evolution of this promising field.

The advent of nanotechnology has ushered in new frontiers for tumor diagnosis and treatment. Nanomaterials with excellent biocompatibility have emerged as vital players in NSCLC imaging, diagnosis, drug delivery, and controlled drug release. These nanomaterials have effectively addressed some of the limitations associated with traditional diagnostic methods, including rapid detection of nucleotide mutations, enhanced tumor marker identification, and improved efficacy of magnetic resonance imaging (MRI) and endobronchial optical coherence tomography (EB-OCT). Notably, molecular beacon-based liposomes, specifically designed to detect AIMP2-DX2 mutations, have immense potential for simplifying and accelerating the detection of nucleotide mutations [35]. Single-walled Carbon Nanotubes (SWNTs) are an appealing choice for detecting biomarkers, such as toluene in the context of NSCLC [36]. Meanwhile, Gold Nanoparticles (AuNPs), Mesoporous Silica Nanoparticles (MSNs), and Carbon Nanostructures stand out as promising optical imaging agents for MRI due to their remarkable emission intensity, unparalleled photostability, and excellent water solubility, among other advantageous properties [37]. Furthermore, Endobronchial Optical Coherence Tomography (EB-OCT) holds significant importance in lung cancer diagnosis and is well-suited for clinical applications. The integration of EB-OCT with nanomaterials represents a promising avenue for early screening and diagnosis, potentially revolutionizing the detection and management of lung cancer.

Nanomaterials have numerous advantages in various therapeutic modalities for NSCLC, including radiotherapy, chemotherapy, targeted therapy, and surgery. For instance, nanomaterials such as Gold Nanoparticles (AuNPs) act as effective radiosensitizers, enhancing the local dose and mitigating the heterogeneity of responses within hypoxic and rapidly proliferating tumor regions [38]. Furthermore, their high surface-to-volume ratio allows for efficient assembly of biomolecules or residues, enhancing the specificity of chemical drug complexes in targeted therapy. This not only amplifies the efficacy of nanomaterial-based treatments but also minimizes toxicity to normal cells [39]. Targeted delivery is a pivotal advantage of the nanomaterials. Through intricate design and strategic modifications, nano-drugs exhibit heightened specificity, enhanced bioavailability, diminished cytotoxicity towards healthy tissues, augmented loading capacity, prolonged half-lives, and tailored drug release profiles. These attributes facilitate the circumvention of the limitations inherent to conventional chemotherapy approaches [40]. In addition, nanomaterials are invaluable for lymphatic imaging, offering high spatial and temporal resolution. They provide precise information on lymph nodes and lymphatic vessels during radical tumor resection and lymphaticovenous anastomosis, ultimately minimizing surgical time, enhancing procedural efficiency, and preventing iatrogenic injuries [41].

Furthermore, nanosystems have immense potential as efficient vectors for antigen delivery in NSCLC vaccines. Nanovaccines represent a significant advancement, offering the potential to decrease both the dosage and frequency of immunization required for numerous vaccines. Remarkably, a single dose can elicit rapid and long-lasting immunity by simultaneously activating humoral and cell-mediated immune responses. This not only enhances their effectiveness but also allows convenient storage at room temperature. Advancements in liposome-based NSCLC vaccines have paved the way for further clinical studies. A notable example is Tecemotide (L-BLP25), a liposomal vaccine targeting human epithelial mucin (MUC1) glycoprotein. Immunotherapy can elicit potent antigen-specific T-cell responses. Neelima et al. [42] underscored its ability to induce a dominant Th1 response and MUC1-specific cytotoxic T lymphocytes (CTLs), highlighting its potential in the fight against NSCLC.

There has been a great deal of research concerning the use of nanomaterial products for NSCLC. Nanotechnology developed rapidly during 2014–2024, and only a handful of such nanomedicines have entered clinical practice. For example, although L-BLP25 has many theoretical advantages (providing rapid and long-lived immunity in a single dose, et al.), Butts et al. [43] conducted a phase III clinical trial and observed no statistically significant variation in overall survival rates among patients with unresectable stage III NSCLC when treated with a post-chemoradiotherapy intervention compared to those receiving a placebo.. This apparent contrast does not necessarily imply flaws in vaccine design, patient responsiveness, or experimental conduct. Rather, it may be indicative of the complex interplay of various factors influencing the performance of nanomedicine in vivo, which often diverges significantly from what is observed in vitro. These include, but are not limited to, variations in cell interaction and tissue transport, the low transfer efficiency of nanomaterials to tumors, and our incomplete understanding of the molecular mechanisms underlying the interaction of ions with living cells. These factors can significantly impact the efficacy and biodistribution of nanomedicines within the body. Furthermore, the immune system's clearance of nanomaterials upon entry into the body poses a significant hurdle. The body's innate immune defenses can rapidly recognize and eliminate foreign particles, such as nanomaterials, which can limit their therapeutic window and reduce their effectiveness. Despite these challenges, advancements have been made in the development of nanomaterials for the treatment and diagnosis of NSCLC. However, further research and exploration are still necessary to overcome these obstacles and develop strategies with better therapeutic effects and higher biosafety. A deeper understanding of the biological barriers and degradation mechanisms that affect nanomaterial performance in vivo could lead to the design of more effective and stable nanomedicines [44–46].

Our study has several limitations. First, by relying solely on the WoSCC database for publications, there is the potential for an incomplete sweep of the available literature. However, it is important to note that the WoSCC database is one of the most expansive and exhaustive global repositories, and is routinely employed as a source for bibliometric analyses. Consequently, the vast data pool from WoSCC mirrors current research trends and advancements in the field. Second, our selection criteria were limited to studies published in English, potentially excluding valuable contributions from other linguistic contexts.

In summary, Nanomedicine has made remarkable progress in the treatment of lung cancer, thanks to the unique properties of nanoparticles. However, many challenges remain that need to be addressed. Future research directions are worth looking forward to, especially AI-based nanoparticle design, an innovation that could significantly improve therapeutic outcomes. In addition, the exploration of personalized nanomedicine is also in line with the trend of personalized medicine and has great potential. Despite some progress in preclinical studies, there are still obstacles to clinical translation, especially the lack of long-term safety data. Therefore, rigorous toxicity studies and well-designed clinical trials are essential. Collaboration among various stakeholders is also essential. In conclusion, nanomedicine holds great promise in the treatment of NSCLC, but achieving clinical translation and ensuring long-term safety remain key issues. In the future, AI-based nanoparticle design and personalized nanomedicine are expected to bring revolutionary changes to this field.

## Data Availability

The datasets generated during and/or analyzed during the current study are available from the corresponding author on reasonable request.
